# The plastid genome and its implications in barcoding specific-chemotypes of the medicinal herb *Pogostemon cablin* in China

**DOI:** 10.1371/journal.pone.0215512

**Published:** 2019-04-15

**Authors:** Caiyun Zhang, Tongjian Liu, Xun Yuan, Huirun Huang, Gang Yao, Xiaolu Mo, Xue Xue, Haifei Yan

**Affiliations:** 1 Guangdong Food and Drug Vocational College, Guangzhou, China; 2 Key Laboratory of Plant Resources Conservation and Sustainable Utilization, South China Botanical Garden, Chinese Academy of Sciences, Guangzhou, China; 3 College of Life Sciences, South China Agricultural University, Guangzhou, China; 4 Guangdong Provincial Key Laboratory of Applied Botany, South China Botanical Garden, Chinese Academy of Sciences, Guangzhou, China; 5 South China Limestone Plants Research Centre, College of Forestry and Landscape Architecture, South China Agricultural University, Guangzhou, China; National Cheng Kung University, TAIWAN

## Abstract

*Pogostemon cablin* (Blanco) Benth. (Patchouli) is not only an important essential oil plant, but also a valuable medicinal plant in China. *P*. *cablin* in China can be divided into three cultivars (Shipai, Gaoyao, and Hainan) and two chemotypes (pogostone-type and patchoulol-type). The pogostone-type and patchoulol-type are, respectively, used for medicinals and perfumes. In this study, we sequenced and characterized the plastid genomes for all three Chinese cultivars and aimed to develop a chemotype-specific barcode for future quality control. The plastid genomes of *P*. *cablin* cultivars ranged from 152,461 to 152,462 bp in length and comprise 114 genes including 80 protein coding genes, 30 tRNA genes, and four rRNA genes. Phylogenetic analyses suggested that *P*. *cablin* cultivars clustered with the other two *Pogostemon* species with strong support. Although extremely conserved in *P*. *cablin* plastid genomes, 58 cpSSRs were filtered out among the three cultivars. One single variable locus, cpSSR, was discovered. The cpSSR genotypes successfully matched the chemotypes of Chinese patchouli, which was further supported by PCR-based Sanger sequences in more Chinese patchouli samples. The barcode developed in this study is thought to be a simple and reliable quality control method for Chinese *P*. *cablin* on the market.

## Introduction

*Pogostemon cablin* (Blanco) Benth. (Lamiaceae), commonly called Patchouli, is a commercially important plant for its essential oil (patchouli oil). The species has been cultivated widely in China, India, Indonesia, Malaysia, the Philippines, and Singapore [1, 2]. However, information on the natural distribution of *P*. *cablin* is lacking, and its wild populations may be extinct [3–5]. Patchouli oil is an important ingredient in perfume and cosmetics industries because it possesses a fixative property that makes other fragrances longer lasting [4, 6]. *P*. *cablin* is reported as one of the top 20 essential oil yielding plants and is considered to have tremendous economic potential [4].

In addition to the traditional use for perfume, *P*. *cablin* in China is also an important traditional Chinese *materia medica* for dispelling dampness in the middle-energizer, summer heat and dampness, acedia, fullness in the chest, hypochondrium issues, cramps, diarrhea, and so on [7]. Due to its vast cultivation in different localities in China under varying environmental conditions, *P*. *cablin* have evolved diverse morphological characteristics and traits (i.e. Glandular hairs, surface characters of stem and leaves [8], and floral and pollen morphology [9, 10]), and has further been divided into at least three cultivars, *Pogostemon cablin* ‘Shipai’ (hereafter Shipai, Guangzhou, Guangdong Province), *Pogostemon cablin* ‘Gaoyao’ (hereafter Gaoyao, cultivated in Zhaoqing, Guangdong Province), and *Pogostemon cablin* ‘Hainan’ (hereafter Hainan, cultivated in Hainan Province) [11, 12]. The *P*. *cablin* populations from Zhanjiang (Guangdong Province) were formerly treated as *P*. *cablin* ‘Zhanjiang’ (cultivated in Zhanjiang region of Guangdong Province, [12]); however, these *P*. *cablin* accessions were introduced from Hainan Province in the 1960s [11, 12], thus the cultivar has been reclassified as the Hainan cultivar. Currently, the cultivars Shipai, Gaoyao, and Hainan are extensively used in the Chinese commercial market [13].

More than 140 compounds have been isolated and identified from *P*. *cablin* [14]. Among them, the major chemical components (such as pogostone, patchoulol, α- and β-patchoulene) are strongly associated with the biological activities of patchouli oil [14]. Moreover, the content ratios of patchoulol and pogostone of patchouli oil have been an index for quality evaluation of Chinese *P*. *cablin* [7, 15]. Different *P*. *cablin* cultivars exhibit significant differences in quality and bioactive components, as these factors are influenced by climate, soil nutrients, and water in the different locations [16]. Based on their main components of essential oils, *P*. *cablin* in China can be divided into two chemotypes: pogostone-type, with a high content of pogostone and a low content of patchouli alcohol, and patchoulol-type, which has a high content of patchouli alcohol but a low content of pogostone [11, 17, 18]. The cultivars Shipai and Gaoyao are pogostone-type, while the cultivar Hainan is patchoulol-type [18]. Traditionally, the patchoulol-type is mainly used in the perfume industry [17], whereas the pogostone-type cultivars are considered medicinal plants in China. The cultivar Shipai is especially considered as “the authentic herb” for containing the highest content of pogostone [11, 15, 18].

However, the production of pogostone, likely the main effective compound in medicinals, is extremely low, since cultivars Shipai and Gaoyao of the chemotype can be only cultivated in the suburbs of Guangzhou (Guangdong) and Gaoyao (Zhaoqing city, Guangdong), respectively. The cultivation of the authentic herb of Shipai has been severely impacted with the urban area expansion of Guangzhou City [15], and its cultivated area is limited to only 0.067 ha. The cultivation of Gaoyao is also very limited [2]. Currently, the patchoulol-type (Hainan cultivar) is often used as a substitute for the pogostone-type in the commercial market, which decreases the quality of the medicine.

It is morphologically difficult to distinguish the two chemotypes of Chinese *P*. *cablin* in the commercial market. An easy and reliable way to identify the two chemotypes of *P*. *cablin* is crucial but has not yet been developed. Previous studies have shown the potential use of Gas chromatography-mass spectrometry (GC-MS) fingerprints of Chinese *P*. *cablin* (especially patchouli alcohol and pogostone) for quality control [15, 19, 20], but GC-MS fingerprinting is hard in practice because the method is time-consuming and requires relatively complicated phytochemical experiments. Liu et al. [11] indicated that *mat*K and nuclear 18S rRNA could be used to distinguish the two chemotypes, but these markers failed when they were re-sequenced and analyzed more recently by Yao et al. [3].

Plastid genomes have been recently recommended as important “extended barcodes” [21, 22]. Based on the rapid progress of high-throughput sequencing (HTS) technology and PCR-free approaches (such as genome skimming) [23], it is feasible to recover plastid genomes at relatively low concentrations of input DNA (even highly degraded DNA from herbarium specimens, [24]). The use of genome skimming to recover plastid genomes is therefore a useful tool for barcoding herbal materials, since the DNA of herbal materials from markets is always highly degraded.

In this study, we sequenced and assembled the plastid genomes of three Chinese *P*. *cablin* cultivars representing two chemotypes (pogostone-type and patchoulol-type). We aimed to characterize all three plastid genomes to develop specific barcodes for discriminating the two chemotypes of Chinese *P*. *cablin*. The main objective of this study was to provide an accurate method for quality control of the medicinal plants and plant medicines on the market.

## Materials and methods

### Ethics statement

The locations of the field studies are neither private lands nor protected areas. No specific permissions were required for the corresponding locations/activities.

### Plant materials and DNA extraction

In the present study, we collected all three Chinese patchouli cultivars, Shipai (*Pogostemon cablin* ‘Shipai’), Gaoyao (*Pogostemon cablin* ‘Gaoyao’), and Hainan (*Pogostemon cablin* ‘Hainan’) [12]. For the verification experiment, 29 accessions, consisting of 16 patchoulol-type and 13 pogostone-type, were included, and information on chemotypes and localities of the accessions is shown in Table 1. One accession of each cultivar was included in the study because of the vegetative propagation of *P*. *cablin*.

**Table 1:** Accessions of the two genotypes of *Pogostemon cablin* tested using Sanger sequencing.

Population	Location	Latitude	Longitude	Cultivar	N	Chemotype	Genotype	Accession Nos.
SH	Sihui, Zhaoqing, China	23°19′57″N	112°43′51″E	*Pogostemon cablin* ‘Hainan’	11	patchoulol-type	Type A	MK539941
KK	Leizhou, Zhanjiang, China	20°33′05″N	110°03′42″E	*Pogostemon cablin* ‘Hainan’	2	patchoulol-type	Type A	MK539942
YC	Leizhou, Zhanjiang, China	22°04′44″N	111°33′17″E	*Pogostemon cablin* ‘Hainan’	2	patchoulol-type	Type A	MK539943
GY1	Gaoyao, Zhaoqing, China	22°54′27″N	112°27′55″E	*Pogostemon cablin* ‘Hainan’	1	patchoulol-type	Type A	MK539944
GY2	Gaoyao, Zhaoqing, China	22°54′27″N	112°27′55″E	*Pogostemon cablin* ‘Gaoyao’	1	pogostone-type	Type B	MK539945
LT	Liantang, Zhaoqing, China	22°56′57″N	112°27′58″E	*Pogostemon cablin* ‘Gaoyao’	11	pogostone-type	Type B	MK539946
SP	Longdong, Guangzhou, China	23°07′59″N	113°20′09″E	*Pogostemon cablin* ‘Shipai’	1	pogostone-type	Type B	MK539947

### DNA sequencing, assembly, and annotation

The accessions were obtained from the South China Botanical Garden and the Guangdong Institute of Chinese Materia Medica, China. Total genomic DNA was extracted from young leaves using a modified cetyltrimethylammonium bromide (CTAB) method [25].

The plastid genome of the accession from the Shipai cultivar was recovered by long-range PCR enrichment using nine conserved primers [26]. The genome was sequenced on a Miseq sequencing platform (details in [27]). The other two accessions (Gaoyao and Hainan) were sequenced using genome skimming technology [23]. Genome skimming was conducted on the Illumina Genome Analyzer (Hiseq 2500 platform, Illumina, San Diego, CA, USA) at the Beijing Genomics Institute (BGI) in Shenzhen, China.

FastQC 0.11.5 (http://www.bioinformatics.babraham.ac.uk/projects/fastqc/) was used to assess the quality of raw reads, and Trimmomatic [28] was used to remove adapters and filter raw reads. The plastid sequence reads were isolated from the raw reads (including non-plastid DNA, such as the nuclear and mitochondrial DNA) based on all known angiosperm plastid genome sequences. High quality reads of the *P*. *cablin* plastid genomes were initially assembled using SPAdes v3.10.1 [29]. Contigs were aligned with the reference plastome of *Pogostemon yatabeanus* (Makino) Press (KP718618) using the Basic Local Alignment Search Tool (BLAST) (ncbi-blast-2.6.0, ftp://ftp.ncbi.nlm.nih.gov/blast/executables/blast+/LATEST/). According to the reference genome sequence (KP718618), the order and direction of the aligned contigs were determined. Aligned contigs were subsequently manually assembled to construct a preliminary sequence of the *P*. *cablin* plastome.

The resulting assembly genome sequence was used as a reference, to which initial paired-end plastid reads were mapped using Bowtie 2.3.1 [30]. Finally, we obtained the consensus sequence as the complete plastid genome of *P*. *cablin*. Initial gene annotations of *P*. *cablin* were transferred from the published plastome sequences of congeneric *P*. *yatabeanus* (KP718618) using Geneious R9 v9.1.4 (Biomatters Ltd, Auckland, New Zealand). These transferred gene annotations were manually corrected by the translation results and comparisons were made to homologous genes from other sequenced plastid genomes in Lamiaceae. The tRNA genes were verified using ARAGORN [31], with necessary manual adjustment. The annotated GenBank file was used to draw the circular plastome map using OGDraw v1.2 (http://ogdraw.mpimp-golm.mpg.de/) [32].

### Comparative analyses of plastid genomes

The protein-coding genes (PCGs) and non-coding regions (intergenic spacers and introns) were extracted and aligned with MAFFT [33], then they were used to estimate nucleotide variability. We first illustrated a variation using the VISTA Viewer [34] to show the mutation hotspots in the plastid genomes of *Pogostemon*. We then calculated the percentage of nucleotide variability for each molecular region by dividing the numbers of nucleotide substitutions (or indels) by the number of nucleotides of the aligned sequence length.

### Phylogenomic analyses

According to the latest molecular results on Lamiaceae published by Li et al. [35], 14 plastid genomes within Lamiaceae plus two outgroup plastid genomes from other members of Lamiales were downloaded from GenBank (S1 Appendix). The PCGs, intergenic spacers, introns, large single-copy region (LSC), small single-copy region (SSC), and inverted repeat (IR) were individually extracted from all 19 plastid genomes of Lamiaceae (including the three *P*. *cablin* plastid genomes in this study), and were aligned using MAFFT [33]. A phylogenetic analysis based on the maximum likelihood (ML) method was conducted to confirm the phylogenetic position of *P*. *cablin* using RAxML-HPC v8 [36] with the GTR + Γ nucleotide substitution model. ML bootstrapping with 1,000 replicates (RAxML rapid bootstrapping algorithm) was used to estimate branch support.

### Characterization of simple sequence repeats in plastid genomes

Simple sequence repeats (SSRs) in plastid genomes of *P*. *cablin* and its relatives (*P*. *yatabeanus* and *P*. *stellatus*) were detected using MISA [37] with the minimum number of repeat parameters set to ten, six, five, five, five, and five for mono-, di-, tri-, tetra-, penta-, and hexa-nucleotides, respectively.

### Chemotype-specific marker identification and verification

The plastid genomes of all Chinese *P*. *cablin* cultivars were aligned by MAFFT [33]. We examined specific nucleotide variations (such as single nucleotide polymorphisms (SNPs), indels, and cpSSRs) between two chemotypes for chemotype-specific barcodes for these cultivars. To verify the validity of specific barcodes in *P*. *cablin*, we tested the barcodes in all accessions by Sanger sequencing. Primer pairs were then designed using Primer3 software [38] to amplify the specific barcodes using Sanger sequencing. The minimum primer annealing temperature was set to 60 ^o^C, and other settings were maintained at default values. A 25 μL polymerase chain reaction (PCR) reaction mixture was prepared and amplified according to the procedure described by Zhang et al. [39]. PCR reactions were conducted in an ETC811 Thermal Cycler (Eastwin Life Sciences, Inc., Beijing, China). All primer pairs were initially tested for successful PCR amplification in all accessions of *P*. *cablin* on 2% agarose gels. Amplicons with single, clear bands on agarose gels were purified and sequenced in both directions on an ABI3730X sequencer (Applied Biosystems, USA) using the amplification primers. The sequenced genotype for each chemotype was deposited in GenBank (Table 1).

## Results and discussion

### Plastid genome features of *Pogostemon cablin* and two congeneric relatives

**Fig 1:**
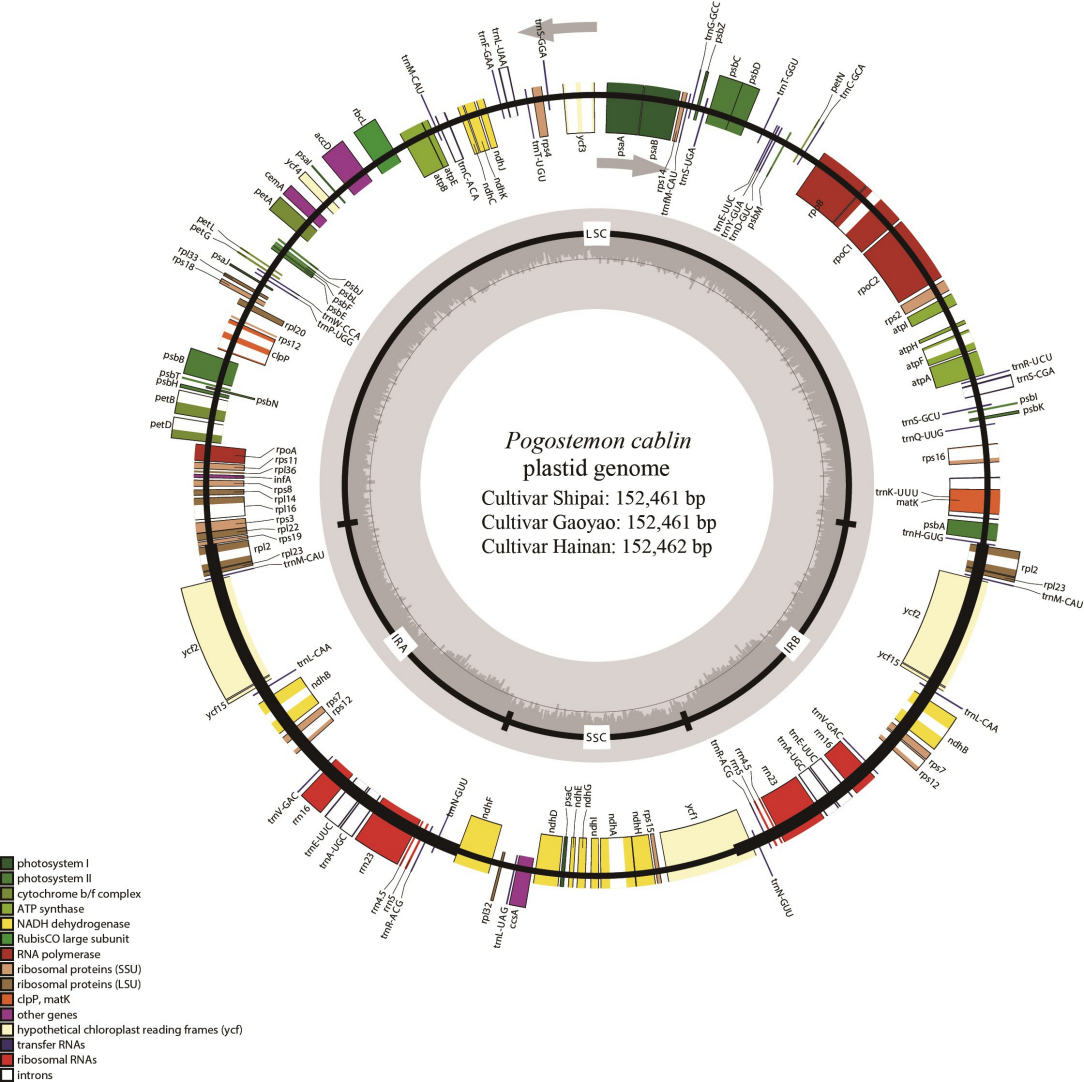
Gene map of the plastid genome of Chinese *Pogostemon cablin* cultivars. Genes outside the circle are transcribed counterclockwise, and genes inside the circle are transcribed clockwise. The gray graph in the inner circle shows the GC content with 50% threshold line.

We obtained *c*. 3 GB paired-end reads for cultivars Hainan and Gaoyao by genome skimming sequencing on the Illumina Hiseq 2500 platform (Illumina, San Diego, CA, USA), and *c*. 2 GB paired-end reads of accession of cultivar Shipai using the Miseq platform. The sizes of the assembled *P*. *cablin* plastid genome are 152,461 bp for Shipai and Gaoyao cultivars, and 152,462 bp for the Hainan cultivar. The complete plastid genomes of *P*. *cablin* with annotations have been submitted to GenBank (accession number MF287372 for Shipai, MF445415 for Gaoyao, and MF287373 for Hainan). All plastid genomes of *P*. *cablin* have the typical quadripartite structure of angiosperm plastid genomes, with a pair of IRa and IRb of 25,662 bp for all cultivars, LSC of 83,553 bp for Shipai and Gaoyao and 83,554 bp for Hainan, and SSC of 17,584 bp for all cultivars (Fig 1). These plastid genomes contain 114 genes, including 80 PCGs, 30 tRNA genes, and four rRNA genes (Table 2). Of these, seven PCGs (*ndh*B, *rpl*2, *rpl*23, *rps*7, *rps*12, *ycf*2, and *ycf*15), seven tRNAs (*trn*A-UGC, *trn*E-UUC, *trn*L-CAA, *trn*M-CAU, *trn*N-GUU, *trn*R-ACG, and *trn*V-GAC), and four rRNAs (*rrn*4.5, *rrn*5, *rrn*16, and *rrn*23) were duplicated in the IRs (S2 Appendix). The overall GC content of the *P*. *cablin* plastid genomes are 38.2%, and the corresponding values of the LSC, SSC, and IR regions are 36.3%, 32.1%, and 43.4%, respectively.

**Table 2:** Features of plastid genomes in five *Pogostemon* taxa.

Species	Size (bp)	LSC (bp)	SSC (bp)	IR (bp)	Number of genes	Number of PCGs	Number of tRNA genes	Number of rRNA genes	Overall GC content (%)	GC content of LSC (%)	GC content of SSC (%)	GC content of IR (%)
*Pogostemon stellatus*	151824	83012	17524	25644	114	80	30	4	38.2%	36.3%	32.1%	43.4%
*Pogostemon yatabeanus*	152707	83791	17568	25674	114	80	30	4	38.2%	36.2%	32.0%	43.4%
*Pogostemon cablin* ‘Shipai’	152461	83553	17584	25662	114	80	30	4	38.2%	36.4%	32.1%	43.4%
*Pogostemon cablin* ‘Gaoyao’	152461	83553	17584	25662	114	80	30	4	38.2%	36.3%	32.1%	43.4%
*Pogostemon cablin* ‘Hainan’	152462	83554	17584	25662	114	80	30	4	38.2%	36.4%	32.1%	43.4%

The plastid genomes of Chinese *P*. *cablin* were compared with the two other published plastid genomes from *P*. *yatabeanus* and *P*. *stellatus* (the only two congeneric relatives with published plastid genomes in GenBank [40]). Comparative analyses of all five plastid genomes of *Pogostemon* showed highly conserved structures and gene organization. The size of *P*. *stellatus* and *P*. *yatabeanus* were 151,825 bp and 152,707 bp, respectively, while the plastid genome sizes of *P*. *cablin* ranged from 152,461 bp to 152,462 bp, depending on the cultivar. The plastid genomes of both *P*. *stellatus* and *P*. *yatabeanus* share the common feature of comprising two copies of IR separated by the LSC and SSC regions, and all contain 80 protein-coding genes, 30 tRNA genes, and four rRNA genes [40]. The two subgenera of *Pogostemon* were separated during the middle to late Miocene (*c*. 15 Mya, [3]), which probably result in small differences in plastid genome structures and their sizes among all five plastid genomes.

### Mutation hotspots in plastid genomes of *Pogostemon cablin*

**Fig 2:**
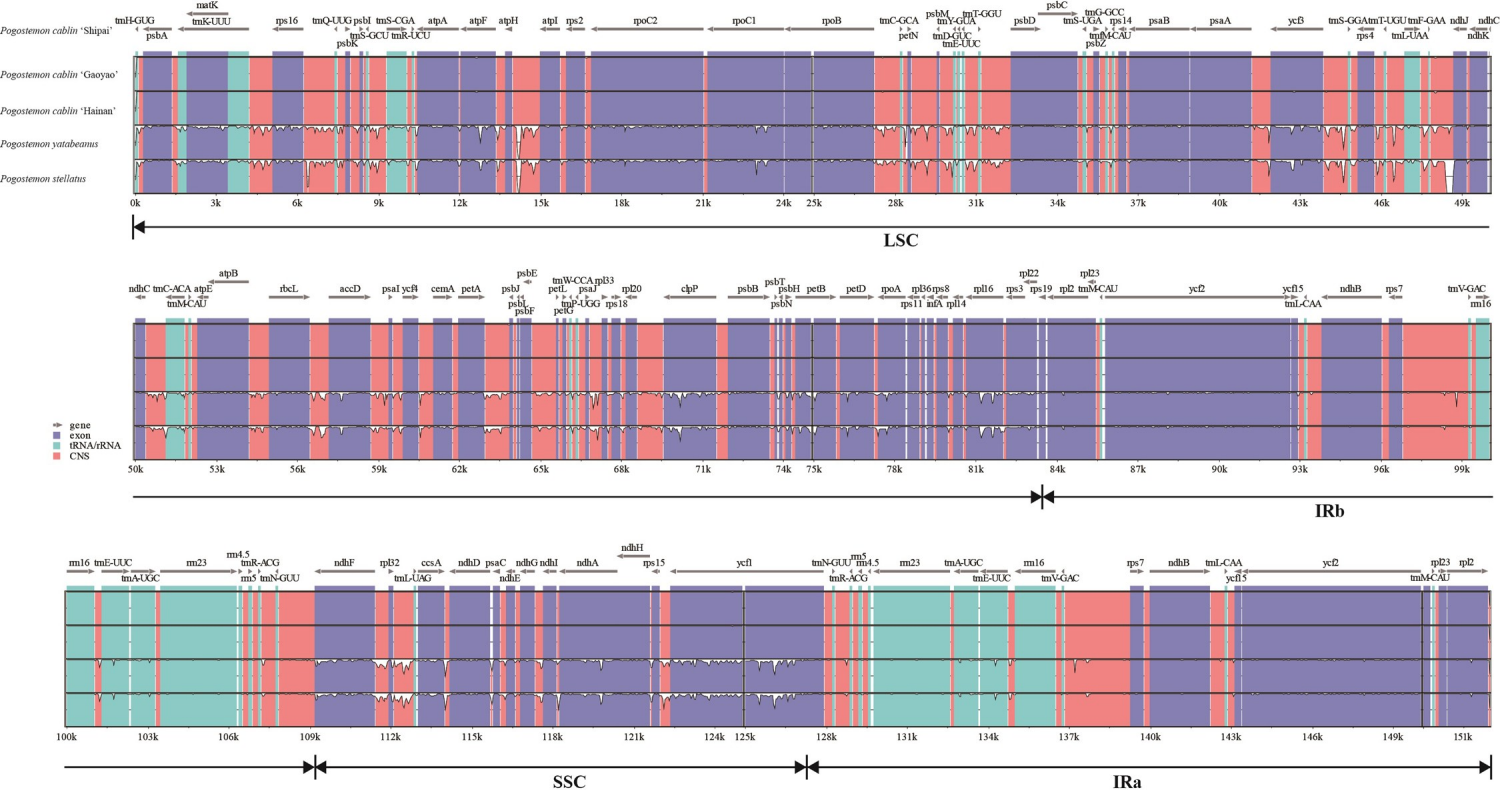
VISTA percent identity plot of five *Pogostemon* plastid genomes with the cultivar *Pogostemon cablin* ‘Shipai’ as a reference.

The distribution of introns and intergenic spacers in the plastid genomes of *Pogostemon* were carefully examined in this study. In total, we identified 21 introns and 100 intergenic spacers. Nine protein-coding genes and six tRNAs contained the single intron, while three protein-coding genes possessed two introns (S2 Appendix). The plastid sequence variations in *Pogostemon* were visualized using VISTA (Fig 2). As expected, the coding and IR regions were more conserved than the non-coding regions and single-copy regions, respectively.

**Fig 3:**
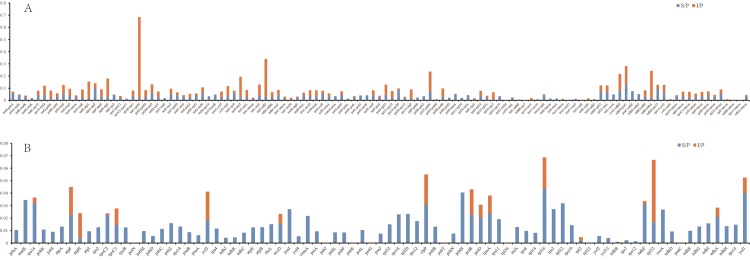
Percentage of variable characters in five aligned plastid genomes of *Pogostemon*. (A) noncoding region; (B) coding region. S/P means the percentage of Single Nucleotide Polymorphisms for each molecular region; I/P indicates the percentage of Indels for each molecular region.

The mutation hotspots in the plastid genomes of *Pogostemon* were further plotted using the proportion of variable sites of coding and non-coding regions. In this analysis, we calculated the percentage of nucleotide variability for each molecular region by dividing the numbers of nucleotide substitutions (or indels) by the number of nucleotides of the aligned sequence length. Specifically, we termed S/P for the percentage of Single Nucleotide Polymorphisms (SNPs) for each molecular region, and I/P for the percentage of Indels (Fig 3). Among the non-coding regions, the average percentage variability was 0.034 for SNPs and 0.045 for indels in intergenic spacers, while these numbers were 0.022 and 0.019 in introns, respectively. The average percentage variability of introns was less than that of the intergenic spacers, which is congruent with the findings of Shaw et al. [41].

The percentage variability of coding regions (introns in coding regions were included herein) were estimated as above. Among the SNPs of coding regions, the percentage variability ranged from 0 (i.e., *pet*N, *psb*J, *psb*E, *pet*G, *psb*T, *rpl*36, *rpl*23, and *psa*C) to 0.044 (*rpl*16), and was 0.014 on average (Fig 3). For the indels of coding regions, the percentage variability ranged from 0 to 0.05 with an average of 0.003. Only seven coding regions without introns had indels (i.e., *rpo*C2, *ndh*F, *rpl*32, *atp*H, *rpo*A, *acc*D, and *ycf*1). Considering the limitation of Sanger sequencing, fragments 500–1,500 bp long is most suitable for Sanger sequencing. We therefore set a strategy for molecular marker selection, of which fragment sizes should range from 500 to 1,500 bp, and the percentage variabilities should be larger than average. Twenty-five highly variable regions, consisting of 14 noncoding regions and 11 coding regions, were summarized in S3 Appendix. These molecular regions might be regarded as potential molecular markers for *Pogostemon* species, but additional studies are required in the future to confirm this.

### Phylogenetic position of *Pogostemon cablin*

**Fig 4:**
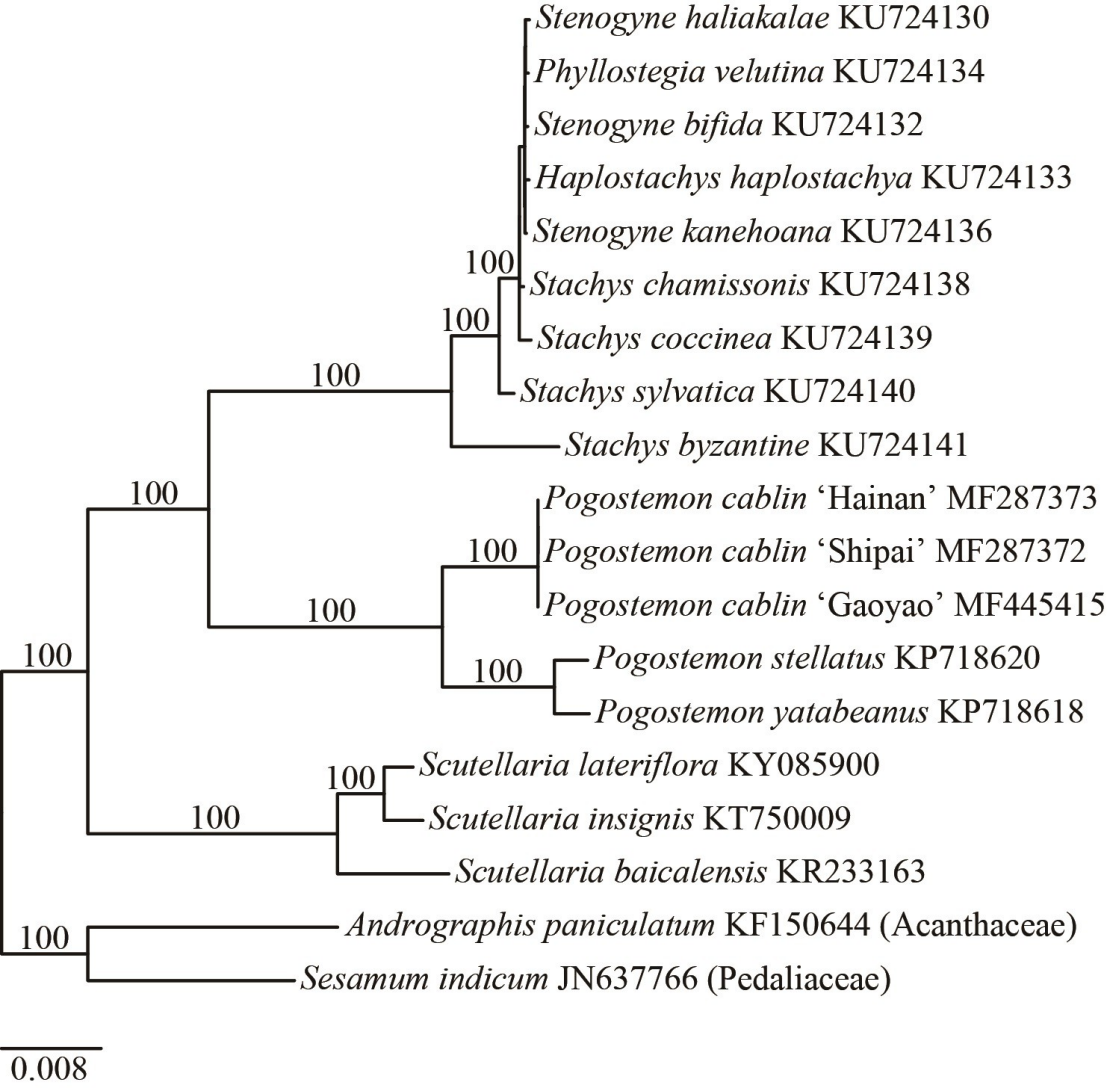
The maximum likelihood tree of Lamiaceae based on the completed plastid genomes. The bootstrap values are given on each main node.

Our phylogenetic trees based on different datasets (i.e., the complete plastid genome, LSC, SSC, IR, CDS, intergenic spacers, and introns) exhibited congruent topologies in *Pogostemon*, which highly supported the monophyly of *Pogostemon cablin* and the two other *Pogostemon* species (Fig 4 and S4 Appendix). As expected, *P*. *yatabeanus* and *P*. *stellatus*, both belonging to subgen. *Dysophyllus*, cluster together with high support (Fig 4). Though our results are congruent with the latest phylogenetic results of *Pogostemon* [3], more *Pogostemon* species (especially the close relatives of *P*. *cablin*) are needed to construct a robust phylogeny and evolutionary history of *Pogostemon* [3].

For over two decades, a driving question in the theory of phylogenetic experimental design has been how to select a set of characters that are evolving at rates appropriate for resolving a given phylogenetic problem [42]. Among the results of all datasets in this study, the ML phylogenies of Lamiaceae in this study were well resolved by the LSC dataset and completed plastid genomes since almost branches were supported with high bootstrap values (S4 and S5 Appendices). Specifically, the phylogenetic relationships in endemic Hawaiian Lamiaceae (a recent radiation group in Hawaii, Welch et al. [43]) could be well resolved using the both datasets (the LSC and completed plastid genomes datasets) in the present study (S5 Appendix). The performance of two datasets in the Lamiaceae showed its potential utility for the construction of a robust *Pogostemon* phylogeny in the future.

### CpSSRs in *Pogostemon cablin* and chemotype-specific markers

Though the rate of molecular evolution in the plastid genome is relatively slow, noncoding plastid DNA can provide informative variation at the species and population level [44]. Because of uniparental inheritance, plastid simple sequence repeats (cpSSRs), which are often located in the noncoding regions of the plastid genome, have the ability to complement nuclear genetic markers in population genetic, biogeographic, and hybridization studies (e.g., [45]).

A total of 58 cpSSRs with lengths of at least 10 bp were detected throughout the *Pogostemon cablin* plastid genomes, including 58 mononucleotides, but no other repeats were found (Table 3). These cpSSR loci were mainly located in intergenic spacers (IGS, 42/58), followed by introns (11/58) and protein-coding regions (5/58). Specifically, five cpSSRs are located in three protein-coding genes (*rpo*C2 (×2), *atp*B, and *ycf*1 (×2)), and eleven are located in seven introns (introns in *trn*K-UUU; *trn*S-CGA, *atp*F (×2), *rpo*C1, *ycf*3, *pet*B (×3), and *rpl*16 (×2)) of the *P*. *cablin* plastid genomes. Most of these SSR loci are found in the LSC region (84.48%), followed by the SSC (12.07%) and IR regions (3.44%). We also listed the cpSSRs of *P*. *yatabeanus* and *P*. *stellatus* in Table 3. Mononucleotide repeats were dominant in these two *Pogostemon* species, and only one and two dinucleotide repeats, respectively, were found in *P*. *yatabeanus* and *P*. *stellatus*. These cpSSRs of all three *Pogostemon* plastid genomes are generally A or T repeats, which is consistent with the AT-richness mainly in intergenic and intron regions of plant plastid genomes [46].

**Table 3:** Statistics of cpSSRs in five *Pogostemon* plastid genomes.

Species	N	LSC	SSC	IRa	IRb	Compound	Mono-(≥10)	Di-(≥6)	A/T	C/G	AT/TA
*Pogostemon cablin* ‘Shipai’	58	49 (84.48)	7 (12.07)	1 (1.72)	1 (1.72)	6 (10.34)	58 (100.00)	No data	57 (98.28)	1 (1.72)	No data
*Pogostemon cablin* ‘Gaoyao’	58	49 (84.48)	7 (12.07)	1 (1.72)	1 (1.72)	6 (10.34)	58 (100.00)	No data	57 (98.28)	1 (1.72)	No data
*Pogostemon cablin* ‘Hainan’	58	49 (84.48)	7 (12.07)	1 (1.72)	1 (1.72)	6 (10.34)	58 (100.00)	No data	57 (98.28)	1 (1.72)	No data
*Pogostemon yatabeanus*	68	55 (80.88)	11 (16.18)	1 (1.47)	1 (1.47)	5 (7.35)	67 (98.53)	1 (1.47)	66 (97.06)	1 (1.47)	1 (1.47)
*Pogostemon stellatus*	62	52 (83.87)	8 (12.90)	1 (1.61)	1 (1.61)	5 (8.06)	60 (96.77)	2 (3.23)	59 (95.16)	1 (1.61)	2 (3.23)

**Fig 5:**
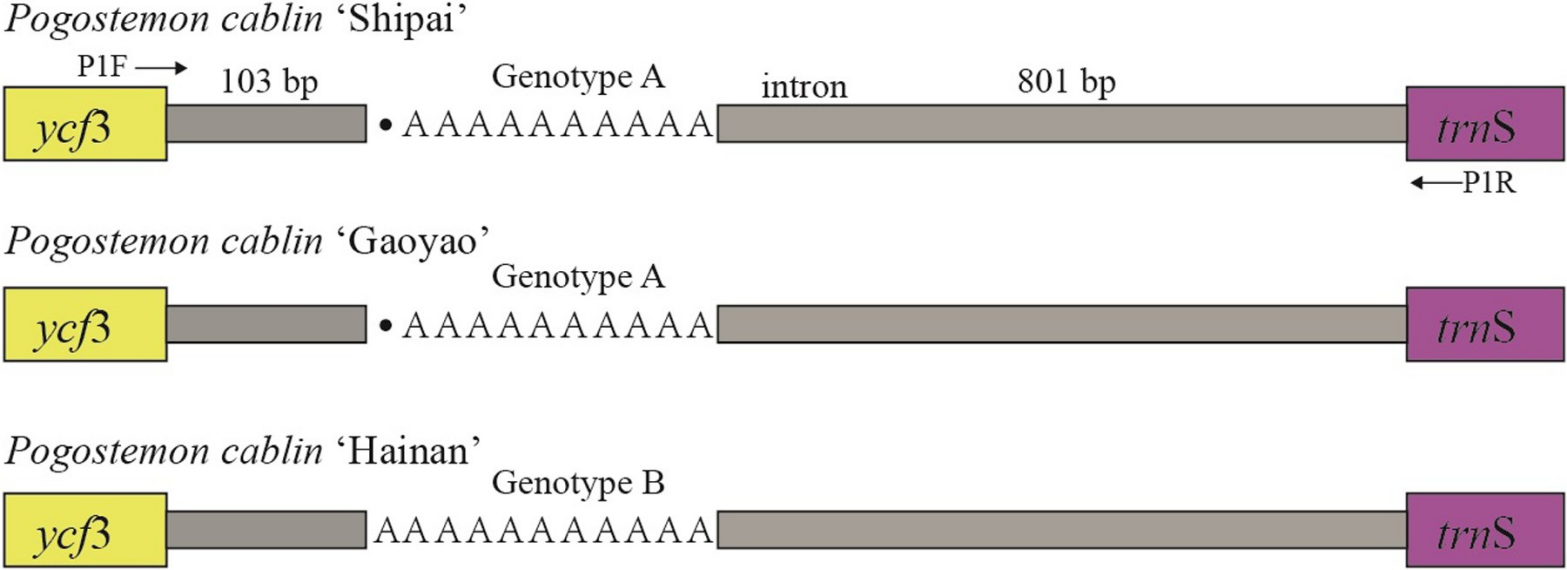
The scaled map of a chemotype-specific locus found in this study.

Since it seldom flowers [9], extensive vegetative propagation is practiced in cultivation [1, 47], resulting in the overall low genetic diversity of *P*. *cablin*. The low genetic diversity has been verified by genome scanning with specific-locus amplified fragment sequencing (SLAF-seq) [18].The previously suggested low mutation rate for the plastid genomes of *P*. *cablin* is supported by this study, since no nucleotide polymorphisms and indels were found among the plastid genomes of all three Chinese cultivars, except one cpSSR locus. The cpSSR locus is located in the intergenic region between *ycf*3 and *trn*S-GGA (ranging from 43,951 bp to 43,960 (or 43,961) bp), and it exhibited a variation in A/T repeats among the *P*. *cablin* cultivars. It is invaluable that the patchoulol-type (Hainan) showed (A/T)_11_ (which indicates Genotype A herein) in the locus, while the pogostone-type (Shipai and Gaoyao) exhibited (A/T)_10_ (Genotype B herein). Using high-fidelity PCR enzymes (PrimeSTAR Max DNA Polymerase, TAKARA, Beijing), we amplified and sequenced the molecular regions (ranging from 967 bp to 968 bp) throughout all accessions using primer pairs (P1F: TCGCGATCTAGGCATAGCTA, P1R: TTCCAATGCTACGCCTTGAA). The scaled map of the locus with primer positions is illustrated in Fig 5. The results of the PCR amplification and Sanger sequencing (both directions) were congruent with the results of the plastid genomes (Table 1), which confirms the presence of the chemotype-specific marker in Chinese *P*. *cablin*.

Liu et al. [11] reported that these two chemotypes correspond to the genotypes of plastid *mat*K and nuclear 18S rRNA. However, *mat*K and ITS (including partial 18S rRNA) did not have any mutations corresponding to the chemotypes ([3]; the data in this study). The GC-MS fingerprint of Chinese *P*. *cablin* is a reliable and straightforward method for quality control [15, 19, 20], but the experiment is time-consuming and requires a large amount of plant materials. Genome scanning filtered out reliable SNPs in *P*. *cablin*, and the genetic groups of Chinese samples matched the two chemotypes [18]. However, SNP discovery on the whole genome level is not a rapid and efficient authentication method, since using bioinformatics for genotype calling is relatively complicated. Recently, Ouyang et al. characterized 45 EST-based SSR markers of *P*. *cablin*, which might be helpful for fingerprinting Chinese *P*. *cablin* cultivars [48].

To our knowledge, the specific cpSSR marker discovered here is the first simple and robust tool for chemotype-specific identification for practical use. Though PCR-based Sanger sequencing for the cpSSR locus is a viable option using the primer pairs presented in this study, we suggest using high-throughput sequencing, such as genome skimming, to obtain the locus in plastid genomes. SSR is essentially derived from replication slippage [49] and could be aggravated by PCR amplification using normal DNA polymerase. Further, the SSR marker itself is difficult for direct sequencing based on the limits of Sanger sequencing. Genome skimming based on HTS effectively overcomes PCR and sequencing errors by yielding large plastid datasets and is able to obtain plastid genomes from multiple sources of plant DNA from fresh to herbarium specimens [24, 50]. Moreover, rapid advancement of bioinformatics and assembly pipelines facilitate the recovery and assembly of plastid genomes from whole genome sequencing data (such as [51, 52]). Overall, the first step to recover the plastid genome is using genome skimming technology, then the specific cpSSR barcode can be extracted from the sequencing data. The chemotype-specific markers developed in this study are a simple and reliable barcode for the quality control of *P*. *cablin* in China.

## Supporting information

S1 Appendix Samples downloaded from GenBank for phylogenetic analysis in this study. (DOCX)

S2 Appendix Gene contents in five plastid genomes of *Pogostemon*. (DOCX)

S3 Appendix The most variable plastid regions of *Pogostemon* in this study. (DOCX)

S4 Appendix The maximum likelihood tree of Lamiaceae based on six different datasets with the bootstrap values on each node. (A) CDS; (B) intergenic spacers; (C) intron; (D) LSC; (E) SSC; (F) IR.
(PDF)

S5 Appendix The maximum likelihood relationships of the endemic Hawaiian Lamiaceae based on seven different datasets with bootstrap values on each node. (A) CDS; (B) intergenic spacers; (C) intron; (D) IR; (E) LSC; (F) SSC; (G) completed plastid genomes.
(PDF)
