# Supplementary material for: The plastid genome and its implications in barcoding specific-chemotypes of the medicinal herb Pogostemon cablin in China
Source: PLoS One. 2019 Apr 15;14(4):e0215512. doi: 10.1371/journal.pone.0215512 (PMC6464210; doi:10.1371/journal.pone.0215512)
Supplement: S1 Appendix — (DOCX) [file pone.0215512.s001.docx]

**S1 Appendix** Samples downloaded from GenBank for phylogenetic analysis in this study.

| **Scientific name** | **Accession Nos.** |
| --- | --- |
| *Haplostachys haplostachya* | KU724133 |
| *Phyllostegia velutina* | KU724134 |
| *Scutellaria baicalensis* | KR233163 |
| *Scutellaria insignis* | KT750009 |
| *Scutellaria lateriflora* | KY085900 |
| *Stachys byzantine* | KU724141 |
| *Stachys chamissonis* | KU724138 |
| *Stachys coccinea* | KU724139 |
| *Stachys sylvatica* | KU724140 |
| *Stenogyne bifida* | KU724132 |
| *Stenogyne haliakalae* | KU724130 |
| *Stenogyne kanehoana* | KU724136 |
| *Pogostemon stellatus* | KP718620 |
| *Pogostemon yatabeanus* | KP718618 |
| *Sesamum indicum* (Pedaliaceae) | JN637766 |
| *Andrographis paniculatum* (Acanthaceae) | KF150644 |
