# Supplementary material for: The plastid genome and its implications in barcoding specific-chemotypes of the medicinal herb Pogostemon cablin in China
Source: PLoS One. 2019 Apr 15;14(4):e0215512. doi: 10.1371/journal.pone.0215512 (PMC6464210; doi:10.1371/journal.pone.0215512)
Supplement: S2 Appendix — (DOCX) [file pone.0215512.s002.docx]

**S2 Appendix Gene contents in five plastid genomes of *Pogostemon.***

| Category | Class | *Pogostemon yatabeanus*  KP718618 | *Pogostemon stellatus*  KP718620 | *Pogostemon cablin*  Cultivar PX | *Pogostemon cablin*  Cultivar ZX | *Pogostemon cablin*  Cultivar NX |
| --- | --- | --- | --- | --- | --- | --- |
| Genetic apparatus | DNA-dependent RNA polymerase | rpoA, rpoB, rpoC1*, rpoC2 | rpoA, rpoB, rpoC1*, rpoC2 | rpoA, rpoB, rpoC1*, rpoC2 | rpoA, rpoB, rpoC1*, rpoC2 | rpoA, rpoB, rpoC1*, rpoC2 |
|  | Maturase | matK | matK | matK | matK | matK |
|  | Large ribosomal subunits | rpl2*(×2), rpl14, rpl16*, rpl20, rpl22, rpl23(×2), rpl32, rpl33, rpl36 | rpl2*(×2), rpl14, rpl16*, rpl20, rpl22, rpl23(×2), rpl32, rpl33, rpl36 | rpl2*(×2), rpl14, rpl16*, rpl20, rpl22, rpl23(×2), rpl32, rpl33, rpl36 | rpl2*(×2), rpl14, rpl16*, rpl20, rpl22, rpl23(×2), rpl32, rpl33, rpl36 | rpl2*(×2), rpl14, rpl16*, rpl20, rpl22, rpl23(×2), rpl32, rpl33, rpl36 |
|  | Small ribosomal subunits | rps2, rps3, rps4, rps7(×2), rps8, rps11, **rps12****(×2), rps14, rps15, rps16*, rps18, rps19 | rps2, rps3, rps4, rps7(×2), rps8, rps11, **rps12****(×2), rps14, rps15, rps16*, rps18, rps19 | rps2, rps3, rps4, rps7(×2), rps8, rps11, **rps12****(×2), rps14, rps15, rps16*, rps18, rps19 | rps2, rps3, rps4, rps7(×2), rps8, rps11, **rps12****(×2), rps14, rps15, rps16*, rps18, rps19 | rps2, rps3, rps4, rps7(×2), rps8, rps11, **rps12****(×2), rps14, rps15, rps16*, rps18, rps19 |
|  | Protease | clpP** | clpP** | clpP** | clpP** | clpP** |
|  | Ribosomal RNAs | rrn4.5(×2), rrn5(×2), rrn16(×2), rrn23(×2) | rrn4.5(×2), rrn5(×2), rrn16(×2), rrn23(×2) | rrn4.5(×2), rrn5(×2), rrn16(×2), rrn23(×2) | rrn4.5(×2), rrn5(×2), rrn16(×2), rrn23(×2) | rrn4.5(×2), rrn5(×2), rrn16(×2), rrn23(×2) |
|  | Transfer RNAs | trnH-GUG, trnK-UUU*, trnQ-UUG, trnS-GCU, trnG-UUC*, trnR-UCU, trnC-GCA, trnD-GUC, trnY-GUA, trnE-UUC, trnT-GGU, trnS-UGA, trnG-GCC, trnfM-CAU, trnS-GGA, trnT-UGU, trnL-UAA*, trnF-GAA, trnV-UAC*, trnM-CAU, trnW-CCA, trnP-UGG, trnH-CAU(×2), trnL-CAA(×2), trnV-GAC(×2), trnI-GAU*(×2), trnA-UGC*(×2), trnR-ACG(×2), trnN-GUU(×2), trnL-UAG | trnH-GUG, trnK-UUU*, trnQ-UUG, trnS-GCU, trnG-UCU*, trnR-UCU, trnC-GCA, trnD-GUC, trnY-GUA, trnE-UUC, trnT-GGU, trnS-UGA, trnG-GCC, trnfM-CAU, trnS-GGA, trnT-UGU, trnL-UAA*, trnF-GAA, trnV-UAC*, trnM-CAU, trnW-CCA, trnP-UGG, trnH-CAU(×2), trnL-CAA(×2), trnV-GAC(×2), trnI-GAU*(×2), trnA-UGC*(×2), trnR-ACG(×2), trnN-GUU(×2), trnL-UAG | trnH-GUG, trnK-UUU*, trnQ-UUG, trnS-GCU, trnS-CGA*, trnR-UCU, trnC-GCA, trnD-GUC, trnY-GUA, trnE-UUC, trnT-GGU, trnS-UGA, trnG-GCC, trnfM-CAU, trnS-GGA, trnT-UGU, trnL-UAA*, trnF-GAA, trnC-ACA*, trnM-CAU, trnW-CCA, trnP-UGG, trnM-CAU(×2), trnL-CAA(×2), trnV-GAC(×2), trnE-UUC*(×2), trnA-UGC*(×2), trnR-ACG(×2), trnN-GUU(×2), trnL-UAG | trnH-GUG, trnK-UUU*, trnQ-UUG, trnS-GCU, trnS-CGA*, trnR-UCU, trnC-GCA, trnD-GUC, trnY-GUA, trnE-UUC, trnT-GGU, trnS-UGA, trnG-GCC, trnfM-CAU, trnS-GGA, trnT-UGU, trnL-UAA*, trnF-GAA, trnC-ACA*, trnM-CAU, trnW-CCA, trnP-UGG, trnM-CAU(×2), trnL-CAA(×2), trnV-GAC(×2), trnE-UUC*(×2), trnA-UGC*(×2), trnR-ACG(×2), trnN-GUU(×2), trnL-UAG | trnH-GUG, trnK-UUU*, trnQ-UUG, trnS-GCU, trnS-CGA*, trnR-UCU, trnC-GCA, trnD-GUC, trnY-GUA, trnE-UUC, trnT-GGU, trnS-UGA, trnG-GCC, trnfM-CAU, trnS-GGA, trnT-UGU, trnL-UAA*, trnF-GAA, trnC-ACA*, trnM-CAU, trnW-CCA, trnP-UGG, trnM-CAU(×2), trnL-CAA(×2), trnV-GAC(×2), trnE-UUC*(×2), trnA-UGC*(×2), trnR-ACG(×2), trnN-GUU(×2), trnL-UAG |
| Light-dependent photosynthesis | Photosystem I | psaA, psaB, psaC, psaI, psaJ | psaA, psaB, psaC, psaI, psaJ | psaA, psaB, psaC, psaI, psaJ | psaA, psaB, psaC, psaI, psaJ | psaA, psaB, psaC, psaI, psaJ |
|  | Photosystem II | psbA, psbB, psbC, psbD, psbE, psbF, psbH, psbI, psbJ, psbK, psbL, psbM, psbN, psbT, psbZ | psbA, psbB, psbC, psbD, psbE, psbF, psbH, psbI, psbJ, psbK, psbL, psbM, psbN, psbT, psbZ | psbA, psbB, psbC, psbD, psbE, psbF, psbH, psbI, psbJ, psbK, psbL, psbM, psbN, psbT, psbZ | psbA, psbB, psbC, psbD, psbE, psbF, psbH, psbI, psbJ, psbK, psbL, psbM, psbN, psbT, psbZ | psbA, psbB, psbC, psbD, psbE, psbF, psbH, psbI, psbJ, psbK, psbL, psbM, psbN, psbT, psbZ |
|  | NAD(P)H dehydrogenase complex | ndhA*, ndhB*(×2), ndhC, ndhD, ndhE, ndhF, ndhG, ndhH, ndhI, ndhJ, ndhK | ndhA*, ndhB*(×2), ndhC, ndhD, ndhE, ndhF, ndhG, ndhH, ndhI, ndhJ, ndhK | ndhA*, ndhB*(×2), ndhC, ndhD, ndhE, ndhF, ndhG, ndhH, ndhI, ndhJ, ndhK | ndhA*, ndhB*(×2), ndhC, ndhD, ndhE, ndhF, ndhG, ndhH, ndhI, ndhJ, ndhK | ndhA*, ndhB*(×2), ndhC, ndhD, ndhE, ndhF, ndhG, ndhH, ndhI, ndhJ, ndhK |
|  | F-type ATP synthase | atpA, atpB, atpE, atpF*, atpH, atpI | atpA, atpB, atpE, atpF*, atpH, atpI | atpA, atpB, atpE, atpF*, atpH, atpI | atpA, atpB, atpE, atpF*, atpH, atpI | atpA, atpB, atpE, atpF*, atpH, atpI |
|  | PS I assembly factor | PafI(ycf3)**, pafII(ycf4) | PafI(ycf3)**, pafII(ycf4) | pafI(ycf3)**, pafII(ycf4) | pafI(ycf3)**, pafII(ycf4) | pafI(ycf3)**, pafII(ycf4) |
|  | Cytochrome b6/f complex | petA, petB*, petD*, petG, petL, petN | petA, petB*, petD*, petG, petL, petN | petA, petB*, petD*, petG, petL, petN | petA, petB*, petD*, petG, petL, petN | petA, petB*, petD*, petG, petL, petN |
| Light-independent photosynthesis | Inner membrane protein | cemA | cemA | cemA | cemA | cemA |
|  | Cytochrome c biogenesis protein | ccsA | ccsA | ccsA | ccsA | ccsA |
|  | Large subunit of Rubisco | rbcL | rbcL | rbcL | rbcL | rbcL |
| other | Acetyl-coA carboxylase subunit D | accD | accD | accD | accD | accD |
|  | Function unknown | ycf1, ycf2(×2), ycf15(x2), infA | ycf1, ycf2(×2), ycf15(×2), infA | ycf1, ycf2(×2), ycf15(×2), infA | ycf1, ycf2(×2), ycf15(×2), infA | ycf1, ycf2(×2), ycf15(×2), infA |

Notes:

* represents gene with one intron.

** represents gene with two introns.

“×2” means gene located in IR region.

Gene name in Bold indicates gene with alternative splicing.
