# Supplementary material for: The plastid genome and its implications in barcoding specific-chemotypes of the medicinal herb Pogostemon cablin in China
Source: PLoS One. 2019 Apr 15;14(4):e0215512. doi: 10.1371/journal.pone.0215512 (PMC6464210; doi:10.1371/journal.pone.0215512)
Supplement: S3 Appendix — (DOCX) [file pone.0215512.s003.docx]

**S3 Appendix** the most variable plastid regions of *Pogostemon* in this study.

| Name | SNPs | Indels | Alignment Length | S/P | I/P |
| --- | --- | --- | --- | --- | --- |
| rbcL | 22 | 0 | 1461 | 0.01506 | 0 |
| ndhI | 8 | 0 | 507 | 0.01578 | 0 |
| petD | 25 | 13 | 1240 | 0.02016 | 0.01048 |
| cemA | 15 | 0 | 690 | 0.02174 | 0 |
| atpF | 29 | 29 | 1292 | 0.02245 | 0.02245 |
| petB | 32 | 28 | 1393 | 0.02297 | 0.0201 |
| rpoA | 24 | 15 | 1023 | 0.02346 | 0.01466 |
| ccsA | 26 | 0 | 972 | 0.02675 | 0 |
| rps3 | 18 | 0 | 663 | 0.02715 | 0 |
| rps16 | 37 | 5 | 1151 | 0.03215 | 0.00434 |
| rpl16 | 59 | 34 | 1354 | 0.04357 | 0.02511 |
| atpF-intron | 25 | 27 | 734 | 0.03406 | 0.03679 |
| petB-intron | 26 | 28 | 745 | 0.0349 | 0.03758 |
| clpP-intron | 49 | 48 | 1392 | 0.0352 | 0.03448 |
| trnS-trnG | 24 | 40 | 676 | 0.0355 | 0.05917 |
| rpoB-trnC | 37 | 43 | 996 | 0.03715 | 0.04317 |
| trnT-psbD | 45 | 30 | 1143 | 0.03937 | 0.02625 |
| trnK-rps16 | 36 | 35 | 901 | 0.03996 | 0.03885 |
| atpB-rbcL | 33 | 17 | 795 | 0.04151 | 0.02138 |
| ndhC-trnC | 36 | 34 | 814 | 0.04423 | 0.04177 |
| petA-psbJ | 42 | 28 | 947 | 0.04435 | 0.02957 |
| rps16-trnQ | 56 | 85 | 1174 | 0.0477 | 0.0724 |
| trnE-trnT | 29 | 26 | 576 | 0.05035 | 0.04514 |
| rpl16-intron | 51 | 34 | 946 | 0.05391 | 0.03594 |
| rpl32-trnL | 50 | 41 | 749 | 0.06676 | 0.05474 |
