# Supplementary figures and images for: The plastid genome and its implications in barcoding specific-chemotypes of the medicinal herb Pogostemon cablin in China
Source: PLoS One. 2019 Apr 15;14(4):e0215512. doi: 10.1371/journal.pone.0215512 (PMC6464210; doi:10.1371/journal.pone.0215512)

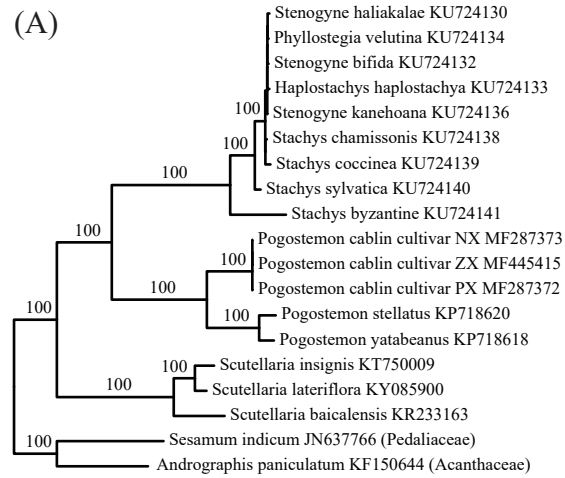

0.006

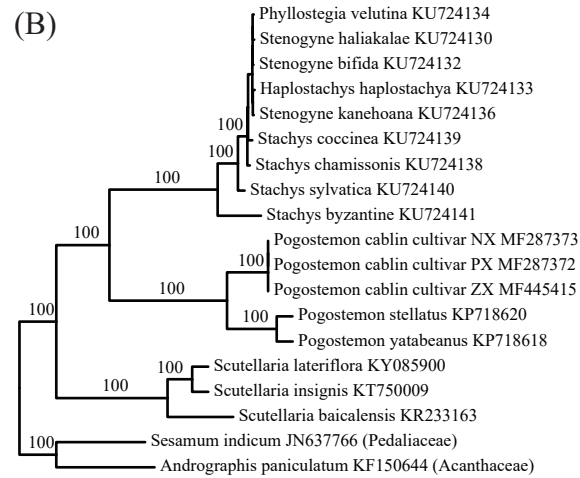

0.02

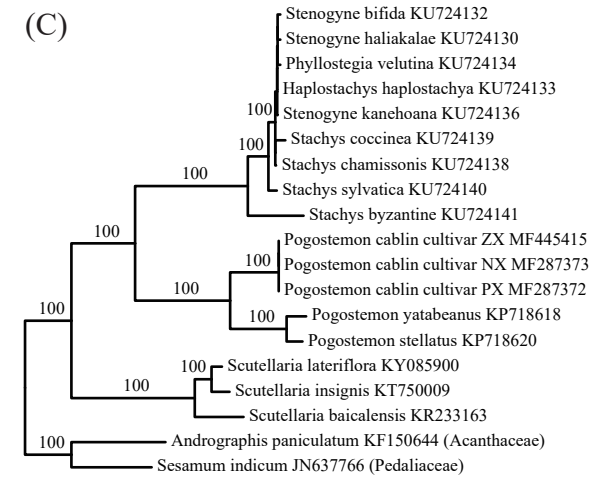

0.008

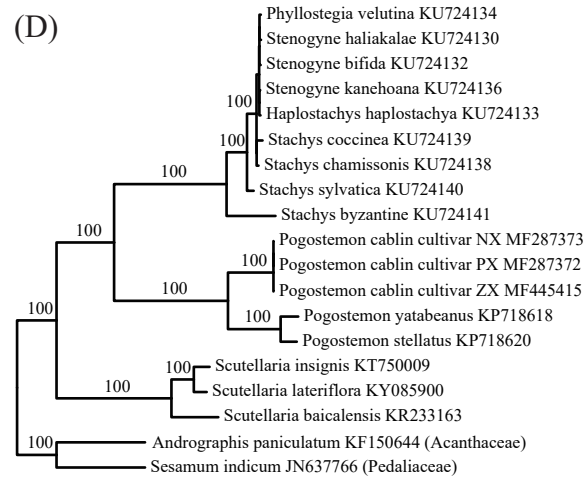

0.009

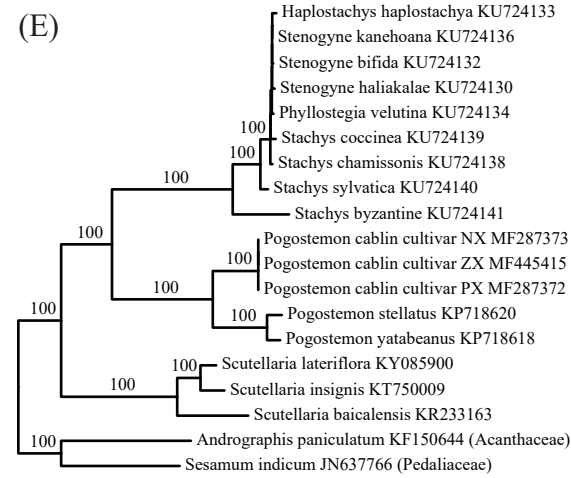

0.02

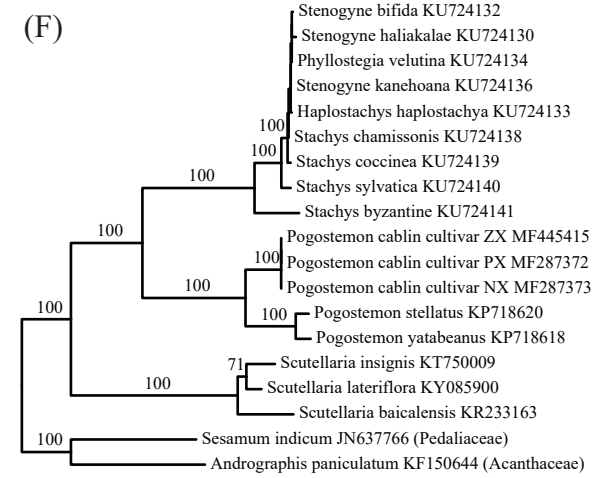

0.002

Supplement: S4 Appendix — (A) CDS; (B) intergenic spacers; (C) intron; (D) LSC; (E) SSC; (F) IR. (PDF) [file pone.0215512.s004.pdf]

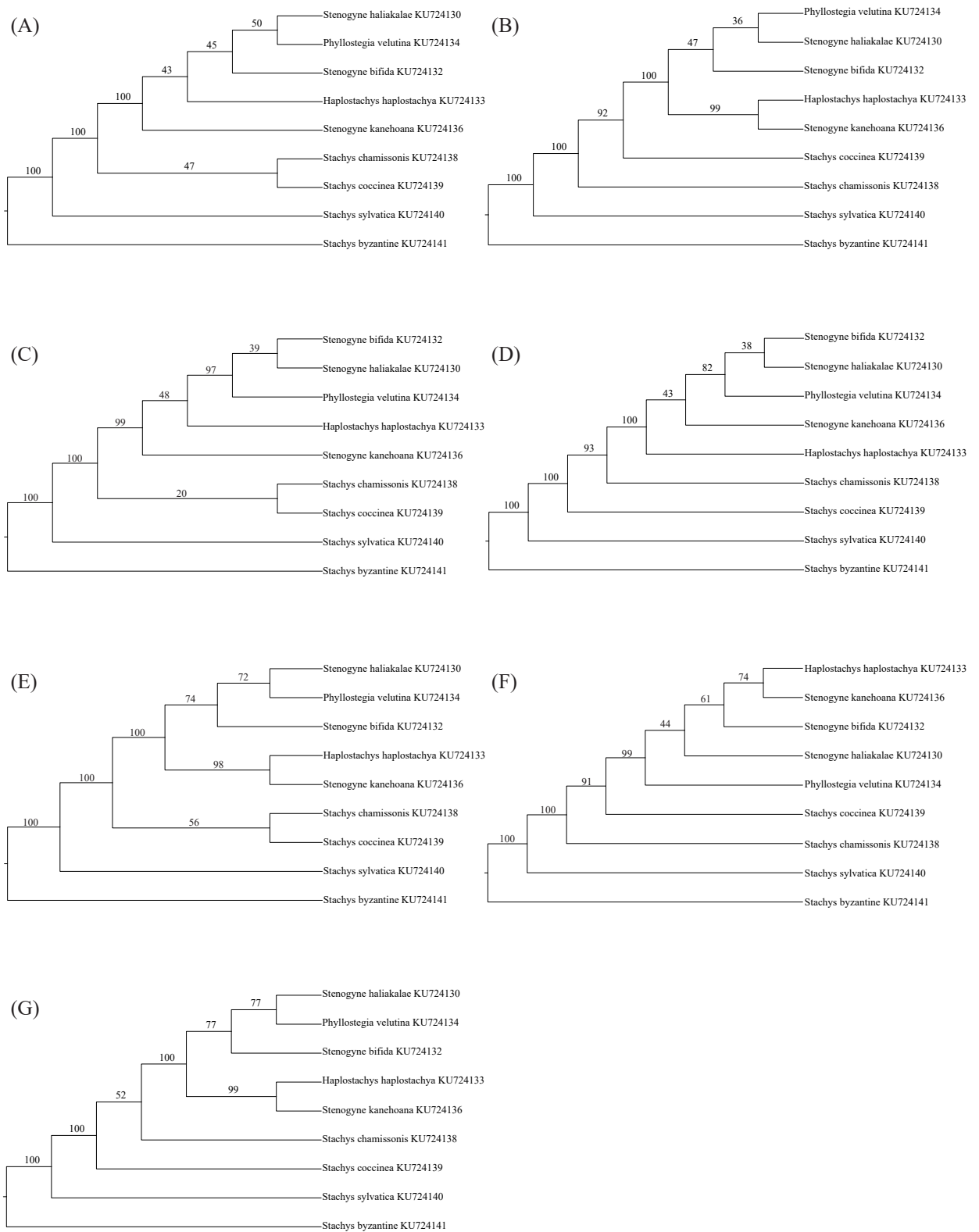

Supplement: S5 Appendix — (A) CDS; (B) intergenic spacers; (C) intron; (D) IR; (E) LSC; (F) SSC; (G) completed plastid genomes. (PDF) [file pone.0215512.s005.pdf]
